# Longitudinal metabolomics in dried bloodspots yields profiles informing newborn screening for succinic semialdehyde dehydrogenase deficiency

**DOI:** 10.1002/jmd2.12075

**Published:** 2020-02-26

**Authors:** Madalyn Brown, Coleman Turgeon, Piero Rinaldo, Ana Pop, Gajja S. Salomons, Jean‐Baptiste Roullet, K. Michael Gibson

**Affiliations:** ^1^ Department of Pharmacotherapy, College of Pharmacy and Pharmaceutical Sciences Washington State University Spokane Washington; ^2^ Mayo Clinic, Department of Laboratory Medicine and Pathology Rochester Minnesota; ^3^ Metabolic Unit, Department of Clinical Chemistry, Amsterdam University Medical Centers Vrije Universiteit Amsterdam, Amsterdam Neuroscience, Amsterdam Gastroenterology & Metabolism Amsterdam The Netherlands; ^4^ Department of Genetic Metabolic Diseases, Amsterdam University Medical Centers University of Amsterdam, Amsterdam Neuroscience, Amsterdam Gastroenterology & Metabolism Amsterdam The Netherlands

**Keywords:** acylcarnitines, amino acids, creatine, dried bloodspots, newborn screening, succinic semialdehyde dehydrogenase deficiency

## Abstract

Analyses of 19 amino acids, 38 acylcarnitines, and 3 creatine analogues (https://clir.mayo.edu) were implemented to test the hypothesis that succinic semialdehyde dehydrogenase deficiency (SSADHD) could be identified in dried bloodspots (DBS) using currently available newborn screening methodology. The study population included 17 post‐newborn SSADHD DBS (age range 0.8‐38 years; median, 8.2 years; 10 M; controls, 129‐353 age‐matched individuals, mixed gender) and 10 newborn SSADHD DBS (including first and second screens from 3 of 7 patients). Low (informative) markers in post‐newborn DBS included C2‐ and C4‐OH carnitines, ornithine, histidine and creatine, with no gender differences. For newborn DBS, informative markers included C2‐, C3‐, C4‐ and C4‐OH carnitines, creatine and ornithine. Of these, only creatine demonstrated a significant change with age, revealing an approximate 4‐fold decrease. We conclude that quantitation of short‐chain acylcarnitines, creatine, and ornithine provides a newborn DBS profile with potential as a first tier screening tool for early detection of SSADHD. This first tier evaluation can be readily verified using a previously described second tier liquid chromatography‐tandem mass spectrometry method for γ‐hydroxybutyric acid in the same DBS. More extensive evaluation of this first/second tier screening approach is needed in a larger population.

**Abbreviations**
C2‐carnitine: acetyl‐carnitine
C4‐OH‐carnitine: 3‐hydroxybutyryl‐carnitine
F: female
M: male

**Synopsis**

Quantitation of ornithine, short‐chain acylcarnitines, and creatine in newborn dried bloodspots may provide a metabolomic profile with potential as an first tier screen for succinic semialdehyde dehydrogenase deficiency.

## 1 INTRODUCTION

Succinic semialdehyde dehydrogenase deficiency (SSADHD) is a rare genetic disease associated with mutations of the ALDH5A1 gene, tissue accumulation of neuromodulators including γ‐aminobutyric acid (GABA) and γ‐hydroxybutyric acid (GHB), and tissue depletion of glutamine (gln), the precursor of GABA and glutamic acid (glu). SSADHD presents with nonspecific mild to moderate developmental delay, intellectual deficiency, severe expressive language impairment, neuropsychiatric problems (ADHD, obsessive compulsive disorder, autistic behavior), and variable epilepsy.^1^, ^2^, ^3^, ^4^, ^5^, ^6^, ^7^, ^8^ There have been reports of sudden unexplained death of epilepsy^9^, ^10^ (Gibson, unpublished).

**Figure 1:**
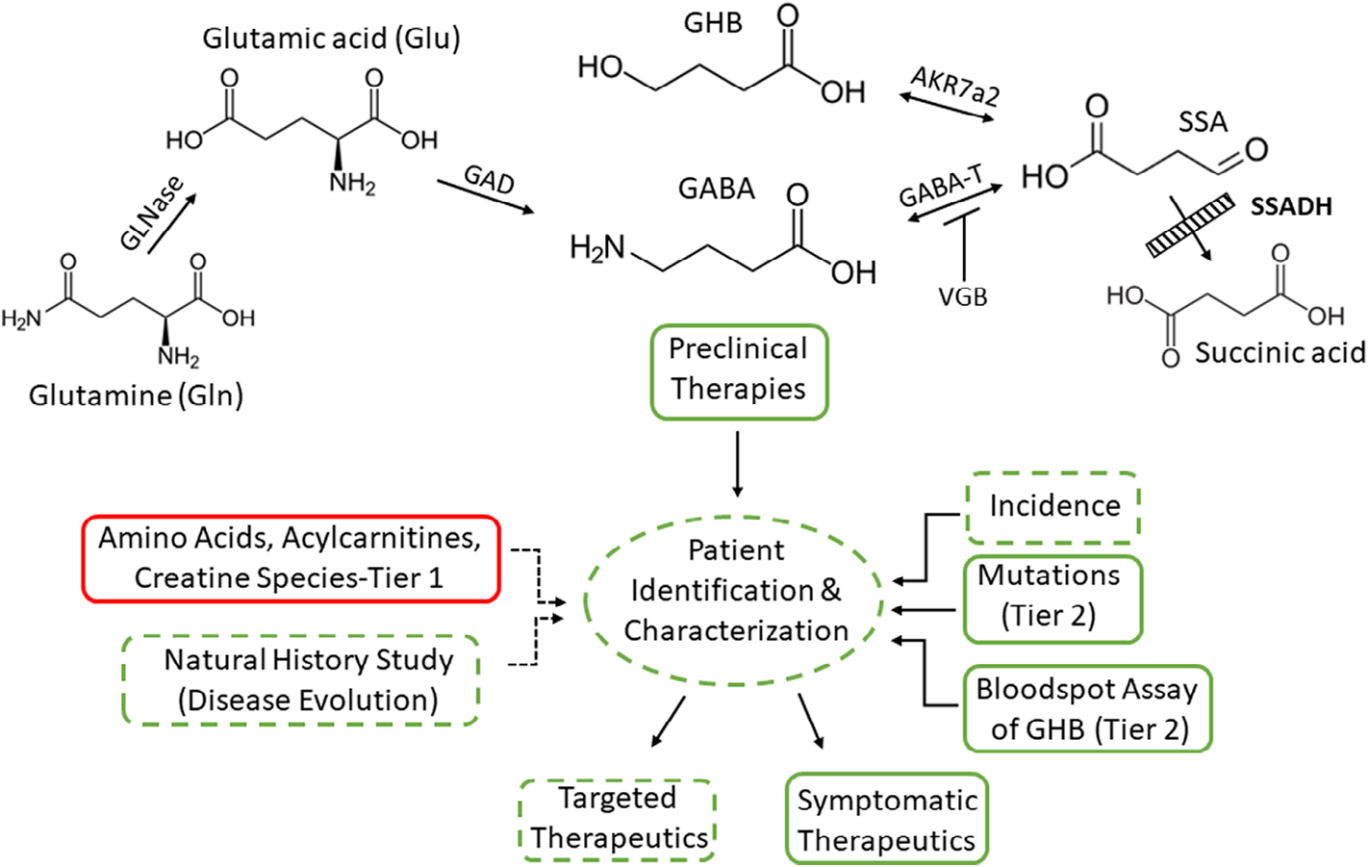
The GABA metabolic pathway (top) and overview of approaches toward patient identification and treatment (bottom). Metabolites elevated in patients with succinic semialdehyde dehydrogenase (SSADH) deficiency (SSADHD) include GABA, SSA (succinic semialdehyde), and GHB (γ‐hydroxybutyric acid); conversely, glutamine (gln) appears decreased. For the diagram, green indicates procedures or measures either achieved (solid line) or partially achieved/in progress (broken line). A tier 1 (tier 2 = confirmation of initial screen) bloodspot assay that is amenable to current NBS platforms is currently required (red). Additional abbreviations: GLNase, glutaminase; GAD, glutamic acid decarboxylase; GABA‐T, GABA‐transaminase (also referred to as ABAT, or aminobutyrate aminotransferase); SSADH, succinic semialdehyde dehydrogenase; AKR7a2, aldo‐keto reductase 7a2. VGB (vigabatrin; γ‐vinylGABA; Sabril) represents an irreversible inhibitor of GABA‐T

Current approaches and major gaps to patient identification and treatment are summarized in Figure 1. Since SSADHD was first described in 1981, research has focused on identifying the spectrum of pathogenic ALDH5A1 mutations, understanding the molecular and biochemical basis of disease presentation, and testing promising therapeutics. The development of an animal model^11^ resulted in significant strides in elucidation of pathomechanisms and development of novel preclinical therapeutics, including a recently completed interventional trial with the GABA_B_R antagonist SGS‐742 (http://www.clinicaltrials.gov; NCT02019667), as well as a completed open‐label trial of taurine which failed to demonstrate efficacy.^12^ Treatment for SSADHD remains symptomatic, yet an expanding therapeutic preclinical pipeline strongly suggests that targeted and effective therapies (in addition to vigabatrin, which directly targets GABA metabolism) for SSADHD are on the horizon.^13^

Implementation of newborn screening (NBS) for SSADHD is clinically important for a number of reasons. Expanded NBS would maximize the therapeutic benefit of targeted therapeutics, facilitate family planning, and remove the potential off‐target effects of commonly used symptomatic agents, effectively constituting “treatment by omission.” Further, NBS could synergize with a recently undertaken natural history study of SSADHD through significant expansion of the span of longitudinal assessment of the disorder. NBS would detect patients who will develop milder forms of the disease, or patients with clinical presentations so subtle that they may be misdiagnosed. The potential for delayed diagnosis is highlighted in the postmortem identification of an adult male in the fifth decade.^5^ Early patient diagnosis would further serve to refine disease prevalence estimates, increase the pool of patients available for a natural history study and future clinical trials, and provide insight on disease pathogenesis and the genetic factors that track with milder disease presentation.

The incidence of SSADHD has been estimated at 1 × 10^6^ based upon mutation (allele) frequency (Dr Nilah Monnier, Stanford‐personal communication). Mutation analysis has been available for many years.^14^ Detection of SSADHD has continued to expand with the addition of ALDH5A1 gene analysis to several commercial epilepsy and intellectual disability panels; however, only ~50% of SSADHD patients have seizures, suggesting that many patients remain undiagnosed. On the other hand, methodology for GHB quantitation in dried bloodspots (DBS) has been presented.^15^ That method utilized liquid chromatography‐tandem mass spectrometry (LC‐MS/MS). At least in the United States, only a very limited number of states employ LC/MS‐MS for metabolite quantification, primarily because it is insufficiently high‐throughput (run time 2‐3 minutes per DBS vs 1 minute with MS/MS alone). States with smaller populations, and an often correspondingly lower birthrate, have the capacity to employ LC‐MS/MS which can expand their metabolic screening “menu,” but states with larger populations (New York, California, and Texas) may only have the capacity to employ LC‐MS/MS as a second tier screen, or for very specialized analytical needs. Nonetheless, GHB quantitation in DBS represents a very attractive second tier test for confirmation of SSADHD.

Recent surveys of SSADHD families worldwide indicate that the median age at disease onset is 1 year whereas the median age at diagnosis is 3 years, that is, a 2‐year delay after the first clinical symptoms. Under current circumstances (with a median age at diagnosis of 3 years), any natural history study of SSADHD would not include patients diagnosed in the early newborn period, representing a major confound from the neurodevelopmental perspective of a disease manifesting a prominent neurological phenotype. Moreover, these survey data provide a *measurable* perspective on the disease burden for SSADHD families who have to wait 3 years to achieve a diagnosis, as well as the public health and societal impact of delayed diagnosis. To address these unmet healthcare needs, we examined the hypothesis that an integrated screen of 19 amino acids, 38 acylcarnitines, and 3 creatine analogues (https://clir.mayo.edu) could be used to potentially identify a metabolomic pattern that could be used as an first‐tier screening tool for SSADHD using DBS, an approach that has been successful in a number of inborn errors of metabolism.^16^, ^17^, ^18^

## 2 MATERIALS AND METHODS

### 2.1 Dried bloodspots

DBS from post‐newborn SSADHD patients were collected with informed consent (WSU IRB 15901). Seventeen post‐newborn DBS included: 10 M/7F, ages 0.8‐38 years (median, 8.2), and 4 sibships (total, 8 patients), representing ~10% of published cases.^19^ SSADHD was previously confirmed through a combination of GHB measurement (urine, DBS), ALDH5A1 molecular analyses and expression, and assay of SSADH in white cells for older patients (Table 1). DBS were obtained using standard finger lance and blood collected onto 903 five spot blood cards (Eastern Business Cards, Greenville, South Carolina). Reference DBS encompassed an archival collection in the Mayo Clinical Laboratories (n = 129‐353, age range 0.5‐87.9 years; mixed gender).

**Table 1:** Characteristics of patients from whom post‐newborn dried bloodspots were obtained

Patient	Age (year)	Gender	Mutation 1	Mutation 2	Zygosity	GHB (physiol. fluid)	Notes on alleles
1	10	F	p.W204*	p.R425*	CH	Elevated	
2	8	M	p.W204*	p.R425*	CH	Elevated	
3	24	M	p.W204*	p.W204*	HZ	Elevated	<10% of nl in white cells
4	40	M	p.W204*	c.1054‐2A>C^a^	CH	Elevated	Undetectable in white cells
5		M	NA	NA			
6	1.5	M	p.G176R	p.G409D	CH	843 mmol/mol (urine; nl < 9)	Both alleles tested in HEK293 overexpression: 0% residual activity^14^
7	26	F	p.G196D	p.G196D	HZ	CSF, 594; sera, 265 μmol/L (nl < 3, both fluids)	Allele p.G196D: 9% residual activity in HEK293 overexpression (Pop et al, unpublished)
8	11	M	See legend	See legend		325 mmol/mol (urine; nl < 9)	
9	7	F	p.T233M	p.T233M	HZ	Elevated	Allele tested in HEK293 overexpression: 4% residual activity^14^
10	15	F	p.T233 M	p.T233M	HZ	Elevated	See above
11	7	M	p.C93F	p.C531Y	CH (parents untested)	477 mmol/mol creatinine (<10)	Allele C93F in HEK293 overexpression: 3% residual activity^14^; C531Y 1% (Pop et al, unpublished)
12	10	M	p.C93F	p.C531Y	CH (parents untested)	114 mmol/mol creatinine (<10)	See above
13	18	F	c.621delC	c.621delC	HZ	Elevated	
14	18	M	p.C93F	p.C93F	HZ	119 mmol/mol (urine; nl < 9)	See above^20^
15	2	F	p.G252C	p.G252C	HZ	Elevated	Allele p.G252C: 6% in HEK293 overexpression (Pop et al, unpublished)
16	5	F	p.G252C	p.G252C	HZ	Elevated	See above
17	8	M	p.G252C	p.G252C	HZ	Elevated	See above

Note: Sibships: patients 2 and 3; 9 and 10; 11 and 12; and 15‐17; all confirmed by sequencing of the parents. For patient 8, Sanger sequencing of ALDH5A1 gene, exon 1 could not be amplified, suggesting a homozygous deletion (MLPA confirmation pending). Control range (nl) for SSADH activity in extracts of white cells, 1.9‐3.9 nmol/min/mg.

Abbreviations: CH, compound heterozygous; HZ, homozygous; NA, not available.

a Splice variant at the canonical splice site which is considered pathogenic.

Newborn DBS were obtained with parental and State Newborn Screening Laboratory consents from seven patients (10 DBS), including three patients providing both first and second screens (approximate age at collection, 48 and 340 hours) (Table 2). Conditions of DBS storage included three first screens stored at 4°C, with the remainder kept at room temperature. Patient overlap between newborn and post‐newborn samples included a single sibship (first, second, and post‐newborn DBS from one sibling, and a second screen [first screen unavailable] with post‐newborn DBS from the older sibling).

**Table 2:** Characteristics of patients from whom newborn dried bloodspots were obtained

Patient	Current age (year)	Gender	Screen 1	Screen 2	Mutation 1	Mutation 2	Zygosity	GHB (urine)
1	10	F		x	p.W204*	p.R425*	CH	Elevated comparable to other patients
2	8	M	x		p.W204*	p.R425*	CH	Elevated comparable to other patients
2	8	M		x	p.W204*	p.R425*	CH	Elevated comparable to other patients
3	4	M	x		p.G533R	c.1015‐2A>C^a^	CH	Elevated comparable to other patients
3	4	M		x	p.G533R	c.1015‐2A>C^a^	CH	Elevated comparable to other patients
4	27	M	x		NA	NA		NA
5	8	F	x		p.M445L	c.610‐2A>G	CH	“Marked elevation”^b^
6	9	M	x		p.W204*	p.G441R^c^	CH	79‐156 mmol/mol
7	5	F	x		c.104_127del p.Ser35*	c.1054‐2A>C	CH	“Marked elevation”
7	5	F		x	c.104_127del p.Ser35*	c.1054‐2A>C	CH	“Marked elevation”

Note: Patients 1 and 2 identical to patients 1 and 2 in Table 1; for urine GHB, control values are <9 mmol/mol creatinine.

Abbreviations: CH, compound heterozygous; HZ, homozygous.

a Splice site SNP [1] at the intron 6/exon 7 junction.^14^, ^21^.

b In addition to elevated 4‐hydroxybutyric acid, the urine organic acids revealed elevations of 4,5‐dihydroxyhexanoic, glutaric, adipic, glycolic, 3‐hydroxypropionic, and 2‐hydroxyglutaric acids, all hallmarks of SSADH deficiency, in two unique urine samples.^22^, ^23^

c PolyPhen characterization of p.G441R indicated a strong likelihood of pathogenicity.

### 2.2 Metabolic measurements

Analyses of amino acids, acylcarnitines (saturated and unsaturated), and creatine derivatives were performed using tandem mass spectrometry as previously described.^18^, ^24^ A single 3 mm punch from one DBS was used. At this time, we do not have insights into specific metabolic profile changes due to prematurity, drug treatment, alimentation, or other external factors which might influence the screening results. This will need to await implemented and expanded NBS for SSADH deficiency.

### 2.3 Collaborative Laboratory Integrated Reports

Collaborative Laboratory Integrated Reports (CLIR 2.12; https://clir.mayo.edu) is a web application that maintains an interactive database of laboratory results from multiple sites and provides on demand clinical decision support for their integrated interpretation.^25^ CLIR replace conventional reference intervals with continuous, covariate‐adjusted moving percentiles,^26^ as well as replacement of analyte decision limits (ie, cutoff values) with a condition‐specific degree of overlap between reference and disease ranges. Further, an additional feature of CLIR incorporates integration of primary markers and unbiased biomarker discovery by automated calculation of all possible permutations of ratios (A/B) plus manual selection of complex ratios and equations, which can be performed simultaneously for all conditions that can be diagnosed from the available laboratory measurements.

### 2.4 Data and statistical analyses

Metabolic measures were integrated within CLIR, facilitating comparison with anonymized DBS data from multiple NBS centers throughout the world. Analyte‐covariate analysis employed age as covariate. Gender was known for reference and patient values, but was only assessed as covariate for informative markers in the post‐newborn DBS samples of SSADHD. Statistical analysis included one‐way analysis of variance or two‐tailed *t* test using GraphPad Prism 8.0 (San Diego, California).

## 3 RESULTS

### 3.1 Informative biomarkers in newborn vs post‐newborn DBS

**Figure 2:**
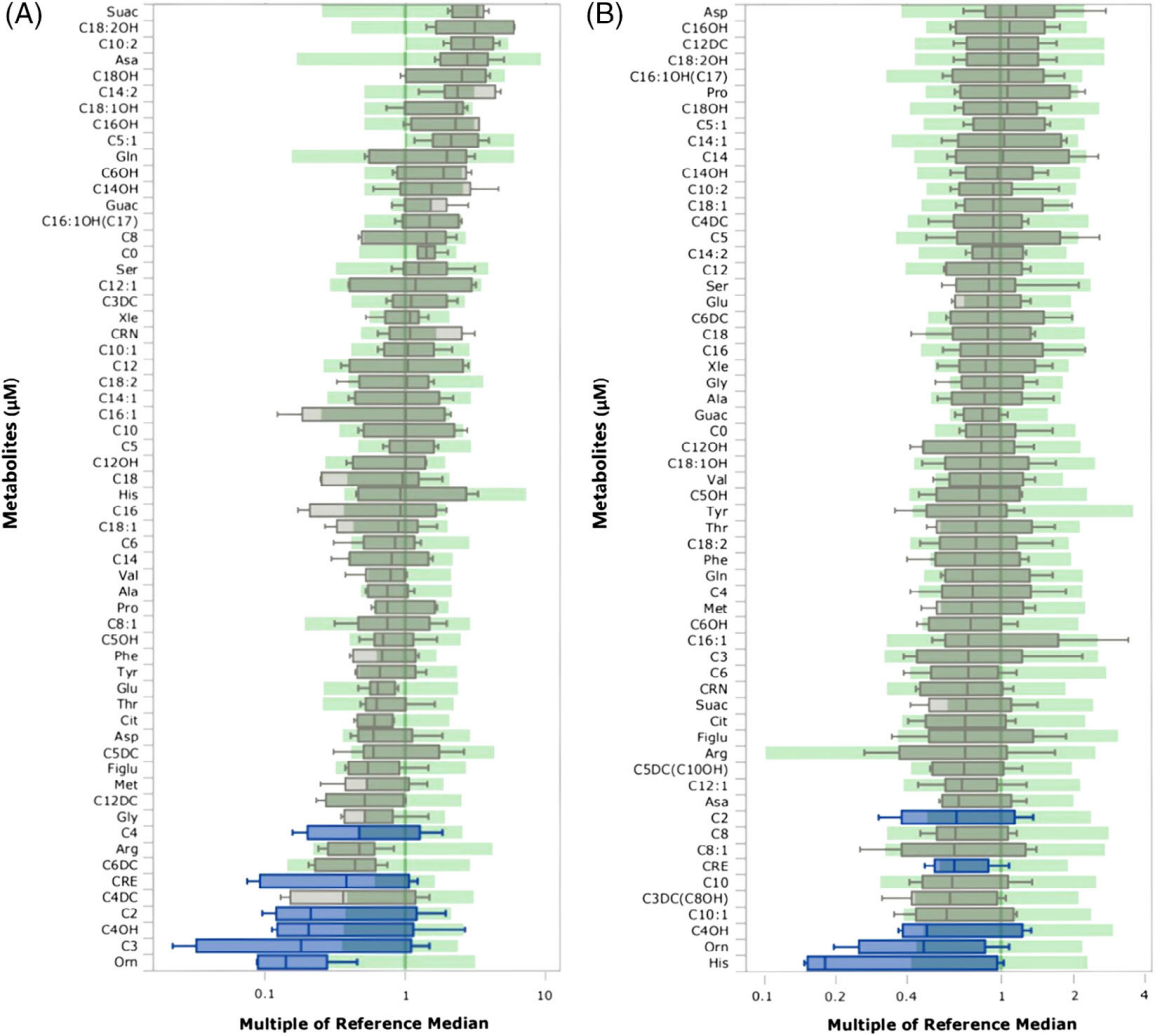
Comparison of multiple analytes against reference intervals for (A) newborn SSADHD DBS and (B) post‐newborn SSADHD DBS. Data for patients is presented as box and whisker (quartiles; 1%ile, 10%ile, median, 90%ile, 99%ile, whereas the reference range (green) lacks whiskers and presents data from 1%ile to 99%ile of reference for that marker. For (A), informative markers (blue) included ornithine, C2‐, C3‐, C4‐ and C4‐OH carnitines, and creatine; for (B), informative markers (blue) included histidine, ornithine, C2‐ and C4‐OH carnitines, and creatine. X‐axis values depict multiple of the reference median, shown for (A) in log scale and for (B) in exponential scale. Amino acids are shown in standard three letter code (eg, his = histidine). Xle represents the sum of leucine and isoleucine, isobaric species. Additional abbreviations: guac, guanidinoacetic acid; C0‐, free carnitine; crn, creatine; suac, succinylacetone; figlu, formiminoglutamic acid. Acylcarnitine metabolites are depicted as chain‐length (eg, C12 = dodecanoylcarnitine), as a monounsaturated (eg, C8:1) or diunsatured (C14:2) species, as the hydroxylated species (eg, C16OH), or as the unsaturated, hydroxylated species (C18:1OH). In selected instances (C16:1OH(C17), C5DC(C10OH), C3DC(C8OH)), identical molecular ions are produced such that the value represents the sum of the two isobaric species shown. DC represents dicarboxylic acid carnitine species (eg, C3DC, malonylcarnitine)

Informative biomarkers are shown in Figure 2 (A, newborn DBS; B, post‐newborn DBS). The *y*‐axis depicts metabolite abbreviations in units of μM. Reference data are shown as green boxes, and depict data falling between the 1%ile and the 99%ile of the reference population for that marker. Patient values are depicted in box and whisker format in quartile ranges. Error bars on the box represent the 1%ile (lowest) and 99%ile (highest) error bar. The bottom of the box represents the 10%ile while the top of the box represents the 90%ile, with the median represented by the horizontal line. The *x*‐axis depicts multiples of reference median in logarithmic scale for newborn reference data (A) and in exponential scale for post‐newborn samples (B). Informative markers (blue) for newborn DBS included C2‐, C3‐, C4‐ and C4‐OH carnitines, ornithine, and creatine (Figure 2A). For post‐newborn DBS, informative markers (blue) included C2‐ and C4‐OH carnitines, ornithine, histidine, and creatine.

The individual data points for Figure 2A,B are comprehensively described in Table 3 (newborn DBS, corresponding to Figure 2A) and Table 4 (post‐newborn DBS, corresponding to Figure 2B). Summary data highlight the mean and SEM (SE of the mean) for the 10 newborn (Table 3) and 17 post‐newborn DBS (Table 4), as well as showing the summary characteristics of data for parallel control DBS available from the CLIR database. For control newborn DBS, shown are the 1st, 50th and 99th centiles for individual control data points ranging from n = 9840 for creatine to up to n = 5 089 207 data points for C3‐carnitine. For control post‐newborn DBS, shown are the 1st and 99th centiles, as well as the median, for individual control DBS data points representing n = 129 for creatine up to n = 353 data points for ornithine.

**Table 3:** Summary of informative markers from newborn dried bloodspots of SSADH‐deficient patients in comparison to control percentiles

Patients	Age (h)	Sex	Ornithine	C2	C3	C4	C4‐OH	CRE
	336	F	12.3	2.7	0.06	0.03	0.033	39.9
	48	M	6.7	3.2	0.08	0.045	0.02	101.4
	360	M	6.6	2.1	0.04	0.048	0.027	30.9
	48	M	10.8	4.9	0.29	0.067	0.047	161.4
	312	M	10.4	3.1	0.19	0.059	0.022	53.3
	48	M	9.4	6.8	0.61	0.196	0.054	216.5
	48	F	19.2	45.2	2.56	0.26	0.50	438.5
	48	M	35.4	24.6	1.76	0.41	0.168	521.0
	336	F	15.6	5.4	0.33	0.188	0.040	159.2
	48	F	9.1	4.7	0.42	0.139	0.033	209.9
Summary		Mean	*13.6*	*10.3*	*0.63*	*0.14*	*0.09*	*193.2*
		SEM	2.7	4.4	0.27	0.04	0.05	52.5
Controls		**Count**	**2 379 498**	**2 935 441**	**5 089 207**	**2 939 759**	**1 065 684**	**9840**
		1%ile	*20.4*	*8.5*	*0.6*	*0.10*	*0.06*	*254*
		50%ile	72	22.9	1.7	0.22	0.18	425
		99%ile	235	47.8	4.1	0.58	0.46	684

Ages (h) are approximate. C2‐, C3‐, C4‐, and C4‐OH represent acylcarnitine species (corresponding to Figure 2A). Additional abbreviations: Count represents the number of newborn data points available from CLIR; CRE, creatine; ile, percentile; SEM, SE of the mean.

**Table 4:** Summary of informative markers in post‐newborn dried bloodspots from SSADH‐deficient patients in comparison to control

Patients	Age (year)	Sex	Ornithine	Histidine	C2	C4‐OH	CRE
	22.7	M	28.9	26.3	5.9	0.06	147.8
	8.7	F	8.8	25.4	4.7	0.04	138.3
	6.8	M	6.3	21.0	4.3	0.06	126.9
	38.2	M	27.1	29.4	12.1	0.07	151.4
	4.1	M	9.4	25.6	7.5	0.05	154.4
	0.9	M	14.2	20.6	17.3	0.15	134.1
	24.3	F	15.7	25.4	7.7	0.05	164.9
	9.6	M	12.1	25.3	6.2	0.07	176.8
	5.8	F	21.9	49.4	9.2	0.06	166.6
	13.5	F	19.1	66.4	10.2	0.05	251.5
	5.6	M	20.8	141.1	9.3	0.05	133.1
	8.2	M	27.1	129.0	9.1	0.05	137.9
	16.6	F	36.9	144.2	12.9	0.14	240.5
	7.1	M	12.5	21.7	9.6	0.04	121.8
	4.2	F	8.8	22.8	9.2	0.05	119.4
	0.8	F	7.6	21.7	15.4	0.11	108.7
	18.4	M	27.6	113.3	19.7	0.15	182.0
Summary		Mean	*17.9*	*53.5*	*10.0*	*0.07*	*156.2*
		SEM	2.2	11.3	1.0	0.01	9.6
Controls		**Count**	**353**	**129**	**350**	**347**	**343**
		Min	13.3	41.0	6.0	0.04	115.2
		Max	88.2	320.3	40.1	0.40	475.2
		Mean	35.3	131.0	15.4	0.13	243.2
		1%ile	*14.4*	*54.8*	*6.9*	*0.05*	*127.8*
		Median	33.4	121.5	14.4	0.12	234.7
		99%ile	72.3	309.6	33.9	0.34	444.8

Control age range, 0.5‐87.9 years, mean 38.3 years. C2‐ and C4‐OH represent informative acylcarnitines (refer to Figure 2B). Additional abbreviations: Count represents the number of control data points available from CLIR; CRE, creatine; ile, percentile; SEM, SE of the mean; min, minimum; max, maximum.

### 3.2 Comparison of gender and screen number for informative markers

Sufficient samples were only available for post‐newborn DBS, and for the informative markers noted above there were no significant differences with respect to gender. For informative markers in newborn DBS, only creatine demonstrated a significant change with age (first screen (~48 hours post birth), [275 ± 68 μM (range 101‐521, n = 6)]; second screen (~312‐360 hours post birth), [71 ± 30 μM (range 31‐159, n = 4); *P* < .05, two tailed *t* test]). This was not unexpected given the inverse age relationship for creatine noted above.

## 4 DISCUSSION

### 4.1 Comparison of metabolic panels to detect SSADHD in DBS

#### 4.1.1 Informative amino acid markers

Based upon the known metabolic correlations between glutamine, glutamate, and GABA,^27^, ^28^, ^29^ our initial prediction was glutamic acid and glutamine would serve as informative markers for SSADHD in DBS. These amino acids were, however, noninformative, whereas ornithine and histidine were. Indeed, the most consistent amino acid dysregulation was that of ornithine, both in post‐newborn and newborn DBS. Low ornithine has hitherto not been reported in plasma amino acid analysis of patients with SSADHD. Conversely, ornithine has been implicated in the ocular toxicity associated with vigabatrin, an antiepileptic whose mode of action encompasses irreversible inactivation of GABA‐transaminase (Figure 1) with concomitant elevation of GABA, a finding analogous to that of SSADHD.^30^ Shank and Campbell^31^ demonstrated that both orn and gln can serve to replenish glu and GABA pools, although gln has a more prominent role in this process.

Of interest, histidine was only informative for post‐newborn DBS and not newborn DBS. It is noteworthy that histidine is conjugated with GABA in CNS to derive the dipeptide homocarnosine, an osmoregulator that is also increased in cerebrospinal fluid of SSADHD patients.^32^, ^33^ Nevertheless, we could not document the presence of homocarnosine in post‐newborn SSADHD DBS, perhaps indicating that the enzyme required for GABA‐histidine conjugation is not active in the newborn period.

#### 4.1.2 Informative acylcarnitine markers

Short‐chain acylcarnitine species were informative in both post‐newborn and newborn SSADHD DBS, encompassing C2‐ and C4‐OH carnitine in the post‐newborn samples and C2‐, C3‐, C4‐, and C4‐OH in newborn SSADHD DBS. This may not be surprising in view of the accumulation of both GHB and succinic semialdehyde in SSADHD,^13^, ^34^ which may interfere with short chain fatty acid metabolism. This observation is further supported by the early reports of dicarboxylic aciduria and unusual tetronic acid derivatives in SSADHD.^22^, ^35^ Depleted levels of acetyl‐carnitine further suggest reduced mitochondrial function, which we and others have observed both in SSADHD and other disorders of fat oxidation.^36^, ^37^ Low levels of 3‐hydroxybutyryl‐carnitine may also provide insight into the success of the ketogenic diet in *aldh5a1* ^−/−^ mice.^38^ Administration of the ketogenic diet to these animals significantly elevated blood levels of 3‐hydroxybutyrate while significantly improving the phenotype of seizures and runted growth in this model.

#### 4.1.3 Creatine as an informative marker for SSADHD DBS

Creatine was an informative marker in both post‐newborn and newborn SSADHD DBS. Previously, we had documented the presence of 4‐guanidinobutyrate in tissue and physiological fluids of both *aldh5a1* ^−/−^ mice and patients with SSADHD.^39^, ^40^ This species is predicted to derive from conjugation of GABA (in lieu of glycine) in the arginine:glycine amidinotransferase reaction of the creatine biosynthetic pathway. On the other hand, if this pathway is engaged, we might expect depletion of arginine in DBS, which was not an informative amino acid marker in either post‐newborn or newborn SSADHD DBS.

#### 4.1.4 Utility of metabolomic profiles as a potential 1st tier screen for SSADH deficiency

For both newborn and post‐newborn SSADHD DBS, there was a degree of overlap with reference ranges. However, the individual markers taken together may provide a metabolomic profile with a high degree of probability for accurate detection of SSADH deficiency. For example, it is informative to compare the mean values of biomarkers for patients (n = 10; Table 3) in comparison to the 1%ile of the control data range (n = 9840‐5 089 207) (the appropriate data are italicized in Table 3). These results highlight the fact that the patient means are below this control percentile for both ornithine and creatine, and very close to this percentile for the four acylcarnitine species. Accordingly, the newborn profile of C2‐, C3‐, C4‐ and C4‐OH acylcarnitines, creatine, and ornithine in DBS may highlight an informative “biosignature” of SSADH deficiency.

For post‐newborn DBS (Table 4), it is similarly informative to compare the mean of patient DBS (n = 17) to the 1%ile of the control DBS (n = 129‐353) for the five biomarkers shown, including orn, his, C2‐ and C4‐OH acylcarnitines, and creatine. For histidine, the mean patient DBS data were below the 1%ile of parallel DBS control data, while the mean C4‐OH for patient DBS is proximal to the parallel 1%ile control DBS data (pertinent data sets in Table 4 are italicized), while the mean for all five biomarkers resides well below the median of control DBS data. The fact that four of six biomarkers show parallel patterns between newborn and post‐newborn SSADHD DBS (ornithine, C2‐ and C4‐OH‐acylcarnitines, and creatine) lends further credence to the idea that this “biosignature” of biomarkers is informative for SSADH deficiency. At this time, and in the absence of specific funding for those studies, retrospective “in silico” analyses of metabolic patients in archival newborn DBS has not been undertaken, but such data will provide key sensitivity and specificity data, as well as predictive value, for the metabolomic profile results we have observed.

## 5 LIMITATIONS AND FUTURE STUDIES

A primary limitation with this study is the low number of both post‐newborn and newborn SSADHD DBS, especially the latter. This issue is compounded by the fact that different states within the USA retain archival NBS DBS for variable times, with some states discarding samples at the end of 1 year, others at 6‐8 years, and still others retaining their samples for decades or indefinitely. This provides inherent challenges in accruing substantial numbers of newborn SSADHD DBS. Moreover, conditions of storage are often variable, with some states storing archival DBS at room temperature, while others may keep these samples at 4°C. For post‐newborn DBS, an additional confound is medication (we did not have information on medication intake for the current study), and the potential influence of medications on DBS metabolites. We plan to significantly expand the number of newborn SSADHD DBS for further evaluation.

It remains to be determined if GABA (free, or esterified) can be quantified in human DBS, but at present this analyte is not present on US NBS panels. Moreover, it would be worthwhile to evaluate the current SSADHD DBS for the presence of 4‐guanidinobutyrate, which may be a potentially relevant biomarker for SSADHD, as well as further characterizing the presence/absence of homocarnosine and carnosine (the latter the dipeptide of his and β‐alanine). These might be pertinent to the development of a sensitive first‐tier screen for SSADHD using DBS. A second‐tier screen already exists that can quantify GHB in DBS (see Introduction^15^, ^41^) or via mutation analysis.^14^ Finally, any methodology pertinent to the newborn detection of SSADHD may have relevance to the identification of GABA‐transaminase deficiency,^42^ as more patients are being identified. With regard the latter, our prediction is that ornithine will remain an informative marker in DBS.

## 6 CONCLUSIONS

Quantitation of short‐chain acylcarnitines, creatine, and ornithine provides a newborn DBS profile with potential as a first tier screening tool for early detection of SSADHD. This first tier evaluation can be readily verified using a previously described second tier LC‐MS/MS method for GHB in the same DBS. Our approach awaits more extensive evaluation in a larger population.

## CONFLICT OF INTEREST

The authors declare no potential conflict of interest.

## AUTHOR CONTRIBUTIONS

Brown, Turgeon, Rinaldo‐data derivation, drafting of manuscript, editing of manuscript.

Pop, Salomons‐data derivation, statistical analyses, manuscript editing, data analysis.

Roullet and Gibson‐preparation of initial manuscript draft, editing, data and statistical analyses.

## ETHICAL APPROVAL STATEMENT

All procedures followed were in accordance with the ethical standards of the responsible committee on human experimentation (institutional and national) and with the Helsinki Declaration of 1975, as revised in 2000. Informed consent was obtained from all families and State Newborn Screening Programs for study inclusion.
